# Floating Ni Capping for High-Mobility p-Channel SnO Thin-Film Transistors

**DOI:** 10.3390/ma13143055

**Published:** 2020-07-08

**Authors:** Min-Gyu Shin, Kang-Hwan Bae, Hyun-Seok Cha, Hwan-Seok Jeong, Dae-Hwan Kim, Hyuck-In Kwon

**Affiliations:** School of Electrical and Electronics Engineering, Chung-Ang University, Seoul 06974, Korea; 18alsrb@naver.com (M.-G.S.); rkdghks95@naver.com (K.-H.B.); ckgustjr0803@naver.com (H.-S.C.); hwanseok518@cau.ac.kr (H.-S.J.); pccdhkim@naver.com (D.-H.K.)

**Keywords:** p-channel SnO, thin-film transistor, floating Ni capping layer, high mobility, bulk channel, percolation conduction

## Abstract

We utilized Ni as a floating capping layer in p-channel SnO thin-film transistors (TFTs) to improve their electrical performances. By utilizing the Ni as a floating capping layer, the p-channel SnO TFT showed enhanced mobility as high as 10.5 cm^2^·V^−1^·s^−1^. The increase in mobility was more significant as the length of Ni capping layer increased and the thickness of SnO active layer decreased. The observed phenomenon was possibly attributed to the changed vertical electric field distribution and increased hole concentration in the SnO channel by the floating Ni capping layer. Our experimental results demonstrate that incorporating the floating Ni capping layer on the channel layer is an effective method for increasing the field-effect mobility in p-channel SnO TFTs.

## 1. Introduction

Nowadays, oxide semiconductor-based thin-film transistors (TFTs) have gained significant attention as the backplane of various displays because of their merits including high mobility, good operational stability, low process temperature, and excellent uniformity [1,2,3,4,5,6]. However, most of oxide-TFT logic circuits were fabricated using only n-channel TFTs because the electrical properties of p-channel oxide TFTs are still much poorer than those of n-channel oxide TFTs [7,8,9,10]. Complementary logic circuits consisting of n- and p-channel transistors have advantages over n-channel logic ones in terms of static power consumption and noise immunity [11,12,13,14,15]; therefore, to use oxide TFTs in more diverse applications, it is crucial to improve the electrical properties of p-channel oxide TFTs. Up till date, various p-type oxide semiconductors of Cu_2_O [16,17], CuO [18,19], NiO [20,21,22], doped ZnO [23], and SnO [24,25,26], have been studied as channel materials for p-channel oxide TFTs. Among these p-type channel materials, SnO has gained special attention; this is because the hybridization of the O 2p and Sn 5_S_ orbitals in the valence-band edge form the pseudo-closed ns^2^ orbitals in SnO, thereby providing an effective hole conduction path [27,28]. However, despite intensive research, most p-channel SnO TFTs reported thus far exhibit low field-effect mobilities (*μ*_FEs_) of ~1–3 cm^2^·V^−1^·s^−1^ [29,30,31,32], thus limiting the development of oxide TFT-based advanced electronic systems. In this study, we fabricated a high mobility p-channel SnO TFT with a *μ*_FE_ of 10.5 cm^2^·V^−1^·s^−1^ utilizing a floating Ni capping layer. Metal or metal-oxide-based floating capping layers have been frequently used to increase the *μ*_FE_ values of various n-channel oxide TFTs [33,34,35,36,37]. However, no study has yet reported the effects of using a metal capping layer in p-channel oxide TFTs. As far as we know, the *μ*_FE_ of 10.5 cm^2^·V^−1^·s^−1^ is the highest value reported in p-channel SnO TFTs to date. Therefore, our experimental results are expected to be widely used in diverse fields requiring high-mobility p-channel oxide TFTs.

## 2. Experimental Procedure

**Figure 1:**
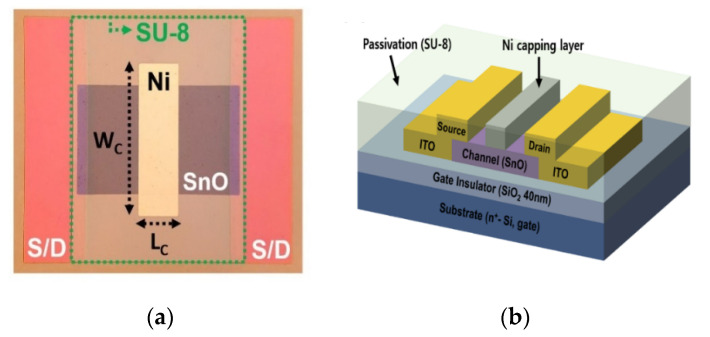
(**a**) Schematic structure; and (**b**) optical microscope image of p-channel SnO TFT with floating Ni capping layer.

Figure 1a,b shows the schematic structure and optical microscope image, respectively, of the p-channel SnO TFT with a floating Ni capping layer. The p-channel SnO TFTs were fabricated on thermal SiO_2_ (40 nm)/highly doped n-type silicon wafer (resistivity < 0.005 Ω·cm), where the highly doped silicon wafer acted as the gate of the TFTs. A 16-nm-thick thin-film was formed using radio frequency (RF) magnetron sputtering with a Sn target (3-inch diameter, 99.999%) without substrate heating in an Ar/O_2_ ambient (Ar/O_2_ = 90 sccm/4 sccm) as a channel layer of the TFTs. The deposition pressure, wafer-to-target distance, and RF power were 3 mTorr, 140 mm, and 60 W, respectively. The thin film deposited on the SiO_2_/n-type silicon wafer was then thermally treated at 180 °C in air ambient for 30 min by using a hot plate [38]. Subsequently, the source/drain electrodes were deposited with 100 nm thick indium-tin oxide (ITO) by using direct current magnetron sputtering; the 70 nm thick Ni floating capping layer was deposited using an e-beam evaporation system. Then, the fabricated devices were subjected to additional thermal treatment at 180 °C for 30 min in air. In this work, Ni was chosen as a material for a floating capping layer because of its high work function and an economical price. Finally, a SU-8 photoresist (thickness: 2 μm) was spin-coated as a passivation layer via the procedure described in our previous paper [39]. A lift-off process was applied to form every layer in this work. The structural properties of the tin oxide thin film were examined by X-ray diffraction (XRD, Rigaku, Tokyo, Japan) with CuKα radiation (λ = 1.5418 Å) at 40 kV and 200 mA. The chemical state and composition of the tin oxide thin film were evaluated by X-ray photoelectron spectroscopy (XPS, Thermo Fisher Scientific, East Grinstead, UK) with Al-Kα source (1486.6 eV) having a 100 μm aperture diameter. The electrical properties of the fabricated SnO TFTs were characterized at room temperature inside the dark chamber using a semiconductor parameter analyzer (Agilent Technologies., Santa Clara, CA, USA).

## 3. Results and Discussion

**Figure 2:**
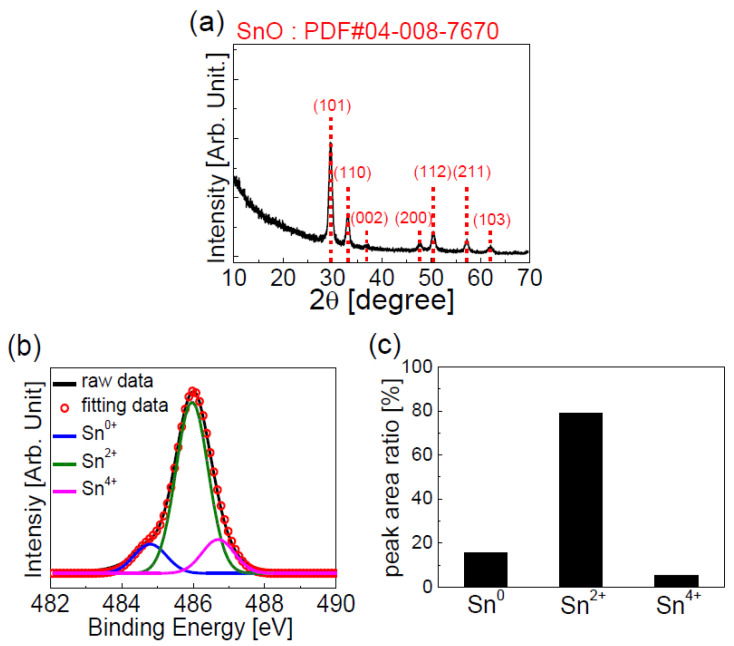
(**a**) XRD pattern; and (**b**) XPS Sn 3d_5/2_3 spectra of tin oxide thin film. (**c**) Relative peak area ratio of Sn^0^, Sn^2+^, and Sn^4+^ components calculated from XPS spectra of tin oxide thin film.

Figure 2a shows the XRD patterns of the thin film formed on the SiO_2_/n-type silicon wafer. We can observe several diffraction peaks from the XRD characterization results, which implies that the thin film is polycrystalline. The XRD patterns in Figure 2a match with the (002), (101), (103), (110), (112), (200), and (211) planes of the tetragonal SnO phase (PDF card number 04-008-7670), which indicates that the dominant phase of the deposited thin film is SnO. Figure 2b shows the XPS Sn 3*d*_5/2_ spectra of the deposited thin film. The XPS spectra were deconvoluted into three sub-peaks stemming from the oxidized states of Sn with 3 different oxidation numbers; here, the binding energies of the Sn^0^, Sn^2+^, and Sn^4+^ components were 484.8, 486.0, and 486.7 eV, respectively [40]. Figure 2c shows the relative peak area ratios of the Sn^0^, Sn^2+^, and Sn^4+^ components calculated from the XPS spectra of the tin oxide thin film in Figure 2b. The XPS characterization results show that the deposited thin film is composed of Sn (15.8%), SnO (78.9%), and SnO_2_ (5.3%); however, Sn^2+^ and SnO are the dominant states/phases.

**Figure 3:**
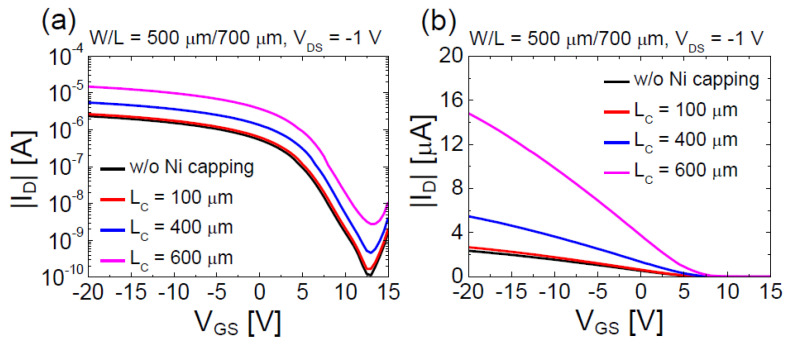
Comparison of (**a**) semi-logarithmic and (**b**) linear scale transfer characteristics of pristine SnO TFT with those of SnO TFTs having different lengths of floating Ni capping layer.

The results in Figure 2c are consistent with the XRD characterization results in Figure 2a. Figure 3a,b compares the semi-logarithmic- and linear-scale transfer characteristics of the pristine SnO TFT with those of the SnO TFTs having different lengths of the floating Ni capping layer, respectively. Here, *I*_D_, *V*_GS_, and *V*_DS_ represent the drain current, gate-source voltage, and drain-source voltage, respectively. The width/length (*W*/*L*) ratio of the channel was 500 μm/700 μm in all TFTs and those of the floating Ni capping layer (*W*_C_/*L*_C_) were 700 μm/100 μm, 700 μm/400 μm, and 700 μm/600 μm. Measurements were conducted at *V*_DS_ = −1.0 V for all TFTs. From the results in Figure 3, it is evident that the floating Ni capping layer enhances the *μ*_FE_ of the SnO TFT, and *μ*_FE_ increases significantly with an increase in *L*_C_. Moreover, we can observe that the threshold voltage (*V*_TH_) and turn-on voltage (*V*_ON_) move slightly toward the positive direction in the SnO TFTs with the floating Ni capping layer compared to the pristine SnO TFT. Table 1 shows the electrical parameters calculated from the SnO TFTs with different *L*c values. Here, *V*_TH_ was extracted from the intercept of the linearly extrapolated curve with the *V*_GS_ axis in Figure 3a and was calculated from the maximum value of the transconductance at *V*_DS_ = −1.0 V using the following equation:

(1) $$\mu_{FE}=\frac{Lg_{m}}{WC_{i}V_{DS}}$$

where *C*_i_ is the capacitance of the gate dielectric per unit area and *g*_m_ is the transconductance. *V*_ON_ is the value of *V*_GS_ at which *I*_D_ increases. The subthreshold swing (*SS*) was calculated using the subthreshold region data in Figure 3b based on the following equation:

(2) $$SS=\frac{dV_{GS}}{d\left(logI_{D}\right)}$$

**Figure 4:**
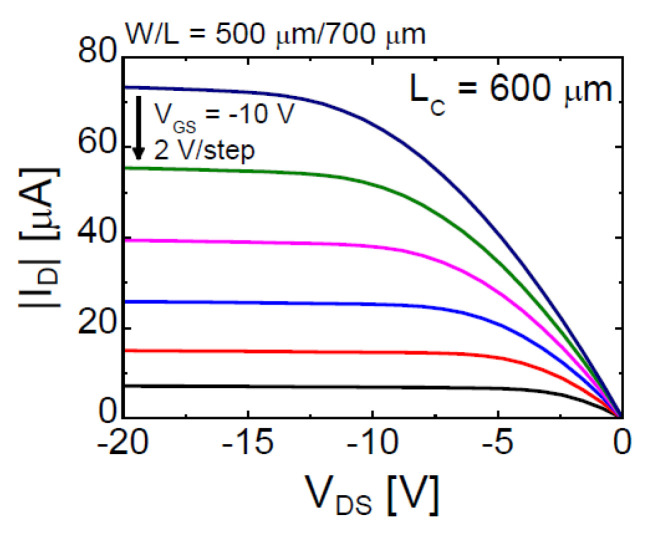
Output characteristic of fabricated p-channel SnO TFT with a floating Ni capping layer (*L_C_* = 600 μm).

**Figure 5:**
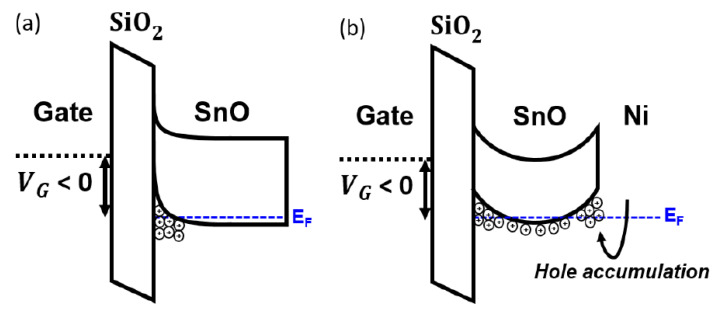
Illustration of band diagrams for (**a**) pristine and (**b**) floating-Ni-capped SnO channels under negative V_GS_.

The data in Table 1 show that the *μ*_FE_ of the SnO TFT increased from 1.7 to 10.5 cm^2^·V^−1^·s^−1^ after incorporating the floating Ni capping layer with *L*_C_ = 600 μm. Figure 4 displays the output characteristics of the floating-Ni-capped SnO TFT (*L*_C_ = 600 μm); the output characteristics show a solid pinch-off and strong saturation behavior. Experimental results in Figure 3 and Figure 4 demonstrate that incorporating of the floating Ni capping layer is an effective method for increasing the *μ*_FE_ value in p-channel SnO TFTs. Nevertheless, the physical mechanism responsible for the increase in *μ*_FE_ after forming the floating Ni capping layer in the p-channel SnO TFT is still controversial. The most plausible mechanism is the changed vertical electric field distribution inside the SnO channel by the floating Ni capping layer. We measured the work-function (*Φ*) of the deposited SnO thin-film as 4.68 eV using the Kelvin probe force microscopy method (Model: KP Technology SKP5050). Considering that Ni has *Φ* value (5.0–5.3 eV) higher than that of SnO [41,42], the back-surface potential of SnO changed such that the holes accumulated at the SnO-Ni interface. Figure 5a,b illustrates the band diagrams for pristine (without capping layer) and floating Ni capped SnO channels, respectively. In the pristine SnO TFT, the negative *V*_GS_ induces hole accumulation only at the front interface (SnO-SiO_2_ interface). However, in the SnO TFT with the floating Ni capping layer, hole accumulation occurs at both front (SnO-SiO_2_) and back (SnO-Ni) interfaces when the negative *V*_GS_ is applied to the gate electrode. Because the channel thickness of the fabricated SnO TFT is very low (t_SnO_ = 16 nm), two channels can overlap and form the bulk channel [42]. When the holes move through the bulk channel, they can avoid scattering at the interface, and *μ*_FE_ can be increased. The increase in *μ*_FE_ caused by the formation of the bulk channel is already reported in previous works for dual-gate IGZO (n-channel) [43] and SnO (p-channel) TFTs [44]. Another possible mechanism is the enhanced percolation conduction caused by the increased hole concentration in the SnO channel. The formation of hole accumulation layers by the floating Ni capping layer could have increased the hole concentration in SnO, which induced an increase in the percolation conduction probability and *μ*_FE_ value [45]. Studies have reported that the random distribution of Sn^2+^ ions modulates the electronic structure of SnO near the valence band maximum and form a potential barrier distribution with a width of a few tens of meV and a height of 0.10 eV [45]. Further studies need to be conducted to understand the exact physical mechanism responsible for the reported phenomenon in this work.

**Figure 6:**
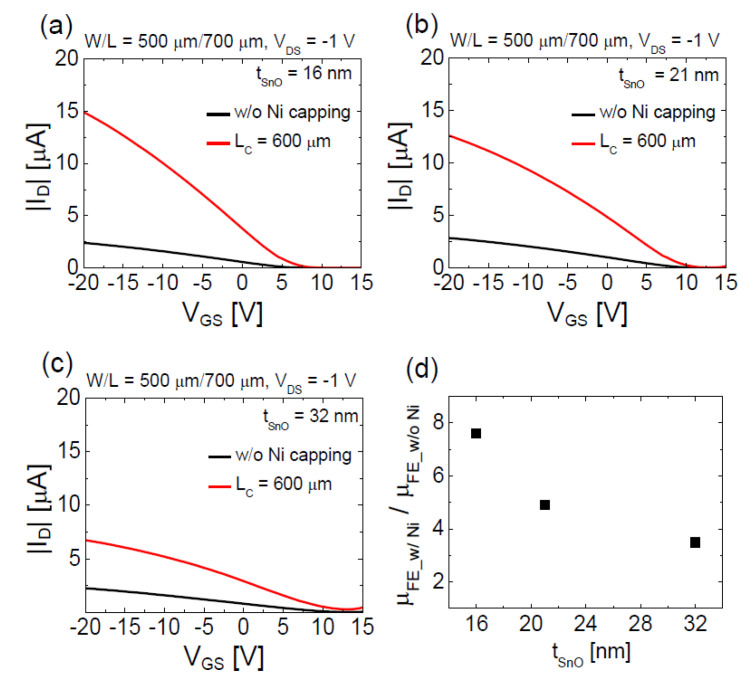
Linear-scale transfer characteristics of p-channel SnO TFTs with and without floating Ni capping layer (*L*_C_ = 600 μm) measured from the devices having different channel thicknesses of: (**a**) 16 nm; (**b**) 21 nm; and (**c**) 32 nm. (**d**) Ratios of the *μ*_FE_ extracted from the SnO TFT with the floating Ni capping layer (*L*_C_ = 600 μm) to that extracted from the SnO TFT without the capping layer in every TFT with different channel thicknesses of 16, 21, and 32 nm. Here, *μ*_FE_w/ Ni_ and *μ*_FE_w/o Ni_ represent *μ*_FE_ extracted from SnO TFT with and without floating Ni capping layer, respectively.

Figure 6a–c shows the linear scale transfer characteristics of the p-channel SnO TFTs with and without the floating Ni capping layer (*L*_C_ = 600 μm) obtained from devices having different *t*_SnO_s of 16, 21, and 32 nm. The data show that *μ*_FE_ of the SnO TFT increases after forming the floating Ni capping layer in all devices; however, the degree of *μ*_FE_ enhancement decreases as the *t*_SnO_ increases. Figure 6d presents the ratios of the *μ*_FE_ extracted from the SnO TFT with the floating Ni capping layer (*L*_C_ = 600 μm) to that extracted from the SnO TFT without the capping layer in every TFT with different *t*_SnO_s.

The results presented in Figure 6 show that the enhanced *μ*_FE_ in the SnO TFT with a floating Ni capping layer is not simply due to the additional conduction path formed by the capping layer, but are consistent with the possible physical mechanisms suggested in the above paragraph because decreases in channel thicknesses facilitate both the bulk channel formation and increase in the channel carrier concentration through band bending at the back-surface in the TFTs.

## 4. Conclusions

In this work, we studied the effects of incorporating a floating Ni capping layer on the electrical characteristics of p-channel SnO TFTs. The obtained results show that the floating Ni capping layer enhanced the *μ*_FE_ of the SnO TFT, and this enhancement became more significant with an increase in *L*_C_ and a decrease in *t*_SnO_. Applying the floating Ni capping layer that almost covered the channel region (*L*_C_/*L* = 600/700 μm) increased the *μ*_FE_ to a value as high as 10.5 cm^2^·V^−1^·s^−1^. Although the physical mechanism responsible for the reported phenomenon is still controversial, the formation of the bulk channel and increase in the percolation conduction probability are considered as possible mechanisms. We believe that our method can offer a simple and promising way to enhance the *μ*_FE_ of p-channel SnO TFTs.

## Figures and Tables

**Table 1:** Electrical parameters measured for the pristine SnO TFT and SnO TFTs with different *L_C_* values.

	w/o Ni Capping	L_c_ = 100 μm	L_c_ = 400 μm	L_c_ = 600 μm
*μ*_FE_ (cm^2^/V·s)	1.7	1.9	4.0	10.5
SS (V/decade)	3.1	2.9	2.8	2.5
V_TH_ (V)	5.2	5.4	5.8	6.1
V_ON_ (V)	12.5	12.6	12.9	13.2

## References

[1] Nomura K., Ohta H., Takagi A., Kamiya T., Hirano M., Hosono H. (2004). Room-temperature fabrication of transparent flexible thin-film transistors using amorphous oxide semiconductors. Nature.

[2] Kamiya T., Nomura K., Hosono H. (2010). Present status of amorphous In-Ga-Zn-O thin-film transistors. Sci. Technol. Adv. Mater..

[3] Lee S.Y., Kim D.H., Chong E., Jeon Y.W., Kim D.H. (2011). Effect of channel thickness on density of states in amorphous InGaZnO thin film transistor. Appl. Phys. Lett..

[4] Kwon J.Y., Jeong J.K. (2015). Recent progress in high performance and reliable n-type transition metal oxide-based thin film transistors. Semicond. Sci. Technol..

[5] Park M.-J., Yun D.-J., Ryu M.-K., Yang J.-H., Pi J.-E., Kwon O.-S., Kim G.H., Hwang C.-S., Bak J.-Y., Yoon S.-M. (2015). Improvements in the bending performance and bias stability of flexible InGaZnO thin film transistors and optimum barrier structures for plastic poly(ethylene naphthalate) substrates. J. Mater. Chem. C.

[6] Noviyana I., Lestari A.D., Putri M., Won M.-S., Bae J.-S., Heo Y.-W., Lee H.Y. (2017). High mobility thin film transistors based on amorphous indium zinc tin oxide. Materials.

[7] Tai Y.-H., Chou L.-S., Chiu H.-L., Chen B.-C. (2012). Three-transistor AMOLED pixel circuit with threshold voltage compensation function using dual-gate IGZO TFT. IEEE Electron. Device Lett..

[8] Kim Y., Kim Y., Lee H. (2013). A novel a-InGaZnO TFT pixel circuit for AMOLED display with the enhanced reliability and aperture ratio. IEEE Electron. Device Lett..

[9] Lin C.-L., Chen P.-S., Lai P.-C., Hsu C.-C., Chang J.-H. (2017). Amorphous IGZO TFT-based pixel buffer to suppress blue-phase liquid crystal high-frequency effect. IEEE Electron. Device Lett..

[10] Chen J.-W., Hu Y.-F., Chen Z.-J., Zhou L., Wu W.-J., Zou J.-H., Xu M., Wang L., Peng J.-B. (2018). A low-power gate driver integrated by IZO TFTs employing single negative power source. Semicond. Sci. Technol..

[11] Nomura K., Aoki T., Nakamura K., Kamiya T., Nakanishi T., Hasegawa T., Kimura M., Kawase T., Hirano M., Hosono H. (2010). Three-dimensionally stacked flexible integrated circuit: Amorphous oxide/polymer hybrid complementary inverter using n-type a-In-Ga-Zn-O and p-type poly-(9, 9-dioctylfluorene-co-bithiophene) thin-film transistors. Appl. Phys. Lett..

[12] Nayak P.K., Caraveo-Frescas J.A., Wang Z., Hedhili M.N., Wang Q.X., Alshareef H.N. (2014). Thin film complementary metal oxide semiconductor (CMOS) device using a single-step deposition of the channel layer. Sci. Rep..

[13] Zhang J., Yang J., He J.-C., Hsu S.-M., Lee C.-C., Su D.-Y., Tasi F.-Y., Cheng I.-C. (2016). Flexible complementary oxide–semiconductor-based circuits employing n-Channel ZnO and p-Channel SnO thin-film transistors. IEEE Electron. Device Lett..

[14] Li Y., Zhang J., Yang J., Yuan Y., Hu Z., Lin Z., Song A., Xin Q. (2019). Complementary Integrated Circuits Based on n-Type and p-Type Oxide Semiconductors for Applications Beyond Flat-Panel Displays. IEEE Electron. Device Lett..

[15] Zhang J., Yang J., Li Y., Wlison J., Ma X., Xin Q., Song A. (2017). High performance complementary circuits based on p-SnO and n-IGZO thin film transistors. Materials.

[16] Zou X., Fang G., Yuan L., Li M., Guan W., Zhao X. (2010). Top-gate low-threshold voltage p-Cu_2_O thin-film transistor grown on SiO_2_/Si substrate using a high-k HfON gate dielectric. IEEE Electron. Device Lett..

[17] Yao Z.Q., Liu S.L., Zhang L., He B., Kumar A., Jiang X., Zhang W.J., Shao G. (2012). Room temperature fabrication of p-channel Cu_2_O thin-film transistors on flexible polyethylene terephthalate substrates. Appl. Phys. Lett..

[18] Sung S.-Y., Kim S.-Y., Jo K.-M., Lee J.-H., Kim J.-J., Kim S.-G., Chai K.-H., Pearton S.J., Norton D.P., Heo Y.-W. (2010). Fabrication of p-channel thin-film transistors using CuO active layers deposited at low temperature. Appl. Phys. Lett..

[19] Maeng W., Lee S.-H., Kwon J.-D., Park J., Park J.-S. (2016). Atomic layer deposited p-type copper oxide thin films and the associated thin film transistor properties. Ceram. Int..

[20] Liu A., Liu G., Zhu H., Shin B., Fortunato E., Martins R., Shan F. (2016). Hole mobility modulation of solution-processed nickel oxide thin-film transistor based on high-k dielectric. Appl. Phys. Lett..

[21] Lee C.-T., Chen C.-C., Lee H.-Y. (2018). Three dimensional-stacked complementary thin-film transistors using n-type Al:ZnO and p-type NiO thin-film transistors. Sci. Rep..

[22] Lin T., Li X., Jang J. (2016). High performance p-type NiOx thin-film transistor by Sn doping. Appl. Phys. Lett..

[23] Lee C.-T., Lin Y.-H. (2014). P-type ZnO thin-film transistors and passivation using photoelectrochemical oxidation method. Appl. Phys. Exp..

[24] Hsu P.-C., Chen W.-C., Tasi Y.-T., Tsai Y.-T., Kung Y.-C., Chang C.-H., Hsu C.-J., Wu C.-C., Hsieh H.-H. (2013). Fabrication of p-Type SnO thin-film transistors by sputtering with practical metal electrodes. Jpn. J. Appl. Phys..

[25] Caraveo-Frescas J.A., Nayak P.K., Al-Jawhari H.A., Granato D.B., Schwingenschlögl U., Alshareef H.N. (2013). Record mobility in transparent p-type tin monoxide films and devices by phase engineering. ACS Nano.

[26] Kim J.-Y., Bae B., Yun E.-J. (2016). Effects of Post-Annealing on the Electrical Properties of Sputter-Deposited SnO Thin-Film Transistors. Sci. Adv. Mater..

[27] Ogo Y., Hiramatsu H., Nomura K., Yanagi H., Kamiya T., Hirano M., Hosono H. (2008). p-channel thin-film transistor using p-type oxide semiconductor, SnO. Appl. Phys. Lett..

[28] Hwang S., Kim Y.Y., Lee J.H., Seo D.K., Lee J.Y., Cho H.K. (2011). Irregular electrical conduction types in tin oxide thin films induced by nanoscale phase separation. J. Am. Ceram. Soc..

[29] Ogo Y., Hiramatsu H., Nomura K., Yanagi H., Kamiya T., Kimura M., Hirano M., Hosono H. (2009). Tin monoxide as an s-orbital-based p-type oxide semiconductor: Electronic structures and TFT application. Phys. Status Solid. A.

[30] Bae S.-D., Kwon S.-H., Jeong H.-S., Kwon H.-I. (2017). Demonstration of high-performance p-type tin oxide thin-film transistors using argon plasma surface treatments. Semicond. Sci. Technol..

[31] Azmi A., Lee J., Gim T.J., Choi R., Jeong J.K. (2017). Performance Improvement of p-Channel Tin Monoxide Transistors with a Solution-Processed Zirconium Oxide Gate Dielectric. IEEE Electron. Device Lett..

[32] Qu Y., Yang J., Li Y., Zhang J., Wang Q., Song A., Xin Q. (2018). Organic and inorganic passivation of p-type SnO thin-film transistors with different active layer thicknesses. Semicond. Sci. Technol..

[33] Zan H.-W., Chen W.-T., Yeh C.-C., Hsueh H.-W., Tasi C.-C., Meng H.-F. (2011). Dual gate indium-gallium-zinc-oxide thin film transistor with an unisolated floating metal gate for threshold voltage modulation and mobility enhancement. Appl. Phys. Lett..

[34] Zan H.-W., Yeh C.-C., Meng H.-F., Tasi C.-C., Chen L.-H. (2012). Achieving High Field-Effect Mobility in Amorphous Indium-Gallium-Zinc Oxide by Capping a Strong Reduction Layer. Adv. Mater..

[35] Kim K.T., Kim J., Kim Y.-H., Park S.K. (2014). In-Situ Metallic Oxide Capping for High Mobility Solution-Processed Metal Oxide TFTs. IEEE Electron. Device Lett..

[36] Choi J.Y., Kim S., Kim D.H., Lee S.Y. (2015). Role of metal capping layer on highly enhanced electrical performance of In-free Si-Zn-Sn-O thin film transistor. Thin Solid Films.

[37] Lee B.H., Sohn A., Kim S., Lee S.Y. (2019). Mechanism of carrier controllability with metal capping layer on amorphous oxide SiZnSnO semiconductor. Sci. Rep..

[38] Cho I.T., Myeonghun U., Song S.H., Lee J.H., Kwon H.I. (2014). Effects of air-annealing on the electrical properties of p-type tin monoxide thin-film transistors. Semicond. Sci. Technol..

[39] Han Y.-J., Choi Y.-J., Cho I.-T., Jin S.H., Lee J.-H., Kwon H.-I. (2014). Improvement of Long-Term Durability and Bias Stress Stability in p-Type SnO Thin-Film Transistors Using a SU-8 Passivation Layer. IEEE Electron. Device Lett..

[40] Jiang Y.-H., Chiu I.-C., Kao P.-K., He J.-C., Wu Y.-H., Yang Y.-J., Hsu C.-C., Cheng I.-C., Chen J.-Z. (2015). Influence of rapid-thermal-annealing temperature on properties of rf-sputtered SnOx thin films. Appl. Surf. Sci..

[41] Bozso F., Arias J.M., Hanrahan C.P., Yates J.T., Metiu H., Martin R.M. (1984). A surface penning ionization study of NH_3_ on Ni (111). Surf. Sci..

[42] Park S., Colombo L., Nishi Y., Cho K. (2005). Ab initio study of metal gate electrode work function. Appl. Phys. Lett..

[43] Xu Y., Liu C., Amegadez P.S.K., Ryu G.-S., Wei H., Balestra F., Ghibaudo G., Noh Y.-Y. (2015). On the origin of improved charge transport in double-gate In-Ga-Zn-O thin-film transistors: A low-frequency noise perspective. IEEE Electron. Device Lett..

[44] Zhong C.-W., Lin H.-C., Liu K.-C., Huang T.-Y. (2015). Improving electrical performances of p-type SnO thin-film transistors using double-gated structure. IEEE Electron. Device Lett..

[45] Qiang L., Liu W., Pei Y., Wang G., Yao R. (2017). Trap states extraction of p-channel SnO thin-film transistors based on percolation and multiple trapping carrier conductions. Solid-State Electron..

